# Cognitive Behavioral Therapy for Insomnia in Geriatric Primary Care

**DOI:** 10.1093/geroni/igab046.1086

**Published:** 2021-12-17

**Authors:** Gregory Hinrichsen, Rosanne Leipzig

**Affiliations:** Icahn School of Medicine at Mount Sinai, New York, New York, United States

## Abstract

Insomnia is common in older adults and may have adverse cognitive, emotional, and physical consequences. Some older people are prescribed sleep medications for insomnia despite longstanding concerns about their use with older people (i.e., BEERS criteria). Cognitive Behavioral Therapy for Insomnia (CBT-I) is highly effective in the treatment for insomnia in adults and older adults. However, most studies of CBT-I in late life have been conducted with individuals younger than 70. This paper discusses four years of experience of providing CBT-I to older people in geriatric primary care, two-thirds of whom were older than 75 years of age. Among the subgroup of 29 individuals who completed a full course of CBT-I, almost all of those who had been on sleep medications discontinued them. Treatment outcomes were large and clinically meaningful. This paper will also describe our experience in providing CBT-I via telehealth because of the COVID pandemic.

